# Familial Hypercholesterolemia in Russia: Three Decades of Genetic Studies

**DOI:** 10.3389/fgene.2020.550591

**Published:** 2020-12-17

**Authors:** Vadim Vasilyev, Faina Zakharova, Tatiana Bogoslovskay, Mikhail Mandelshtam

**Affiliations:** ^1^Institute of Experimental Medicine, Saint Petersburg, Russia; ^2^St. Petersburg State University, Saint Petersburg, Russia

**Keywords:** familial hypercholesterolemia, mutation spectrum, genetic studies in Russia, PCSK9 gene, low-density lipoprotein receptor gene, APOB gene

## Abstract

The first studies of familial hypercholesterolemia (FH) in Russia go back to late 1980-ies. For more than 10 years the research in this field was carried out in Saint-Petersburg, the megapolis in the North-West Russia. Studies were focused on the search for causative mutations in low-density lipoprotein receptor gene (*LDLR*). Gradually the research was spread to Petrozavodsk in Karelia and in the XXI century two more centers contributed in investigations of genetics of FH, i.e., in Moscow and Novosibirsk. The best studied is the spectrum of mutations in *LDLR*, though genetic abnormalities in *APOB* and *PCSK9* genes were also considered. Despite that some 40% mutations in *LDLR* found in Saint-Petersburg and Moscow are referred to as specific for Russian population, and this proportion is even higher in Karelia (ca. 70%), rapid introduction of NGS and intensifying genetic research all over the world result in continuous decrease of these numbers as “Slavic” mutations become documented in other countries. The samplings of genetically characterized patients in Russia were relatively small, which makes difficult to specify major mutations reflecting the national specificity of FH. Moreover, the majority of studies accomplished so far did not explore possible associations of certain mutations with ethnic origin of patients. By now the only exception is the study of Karelian population showing the absence of typical Finnish mutations in the region that borders on Finland. It can be concluded that the important primary research partly characterizing the mutation spectrum in FH patients both in the European and Siberian parts of Russia has been done. However, it seems likely that the most interesting and comprehensive genetic studies of FH in Russia, concerning various mutations in different genes and the variety of ethnic groups in this multi-national country, are still to be undertaken.

## Introduction

At present the term “familial hypercholesterolemia” (FH) has been used to describe different genetic conditions. Traditionally FH is used to designate the monogenic forms of the disease (Defesche et al., 2017), others have proposed also to include polygenic disorders causing elevated plasma cholesterol (Masana et al., 2019). Any application of the term implies the specific elevation of blood plasma low-density lipoprotein (LDL), which increases the risk of cardiovascular incidents (Sarraju and Knowles, 2019).

For example, recent paper has categorized FH into four types (Masana et al., 2019): (1) monogenic heterozygous FH, where patients carry functionally significant mutations in a single allele of the *LDLR* (GeneID: 3949, GenBank NM_000527.4), *APOB* (GeneID: 338, GenBank NM_000384.3), or *PCSK9* (Gene ID: 255738, GenBank NM_174936.4) genes; (2) monogenic homozygous FH, caused either by dominant mutations in *LDLR*, *APOB*, or *PCSK9*, or recessive mutations in the *ARH* (*LDLRAP1*) gene (Gene ID: 100286472, GenBank NM_015627.3), with both alleles affected; (3) polygenic FH, featuring clear manifestations of the clinical phenotype, despite patients having no mutations in genes associated with classical FH, but differing from non-familial cases of multifactorial hypercholesterolemia; and (4) FH combined with hypertriglyceridemia, which includes a subgroup of patients with familial combined hypercholesterolemia (FCH), who satisfy the criteria for FH, but also exhibit hypertriglyceridemia. This classification has primary significance for the choice of therapeutic approach (Masana et al., 2019). This review concerns only monogenic forms of FH as interpreted by Defesche et al. (2017), since genetic testing in Russia was carried out exclusively for these types of the disease. Generally, knowledge of the lipid profile of the patient and their family history of hypercholesterolemia is sufficient for diagnosis of FH. Although not mandatory, genetic testing is helpful for confirming diagnoses (Defesche et al., 2017). Such testing is challenging, given the substantial heterogeneity of the disease. In monogenic FH, causative mutations are most frequently found in the LDL receptor gene, *LDLR*, and patients with such mutations comprise up to 80% of all FH cases. More than 2,000 different mutations in *LDLR* have been documented worldwide, the majority of which are rare, except in populations with strong founder effects. The great majority (50–60%) of nucleotide substitutions in *LDLR* are missense mutations, further increasing the difficulty of DNA-based diagnosis of heterozygous forms of FH, since for each genetic alteration discovered the causal role in the development of hypercholesterolemia must be demonstrated (Mandelshtam, 2011).

Mutations in *APOB* causing FH vary in frequency among populations, and account for roughly 5–10% of all FH cases globally, although the contribution of these mutations may be somewhat higher in Central Europe (Germany, Austria, and Switzerland) (Andersen et al., 2016). Mutations in *PCSK9* and *LDLRAP1* resulting in FH are rare; specifically, mutations in *PCSK9* are responsible for a maximum of 1% of all FH cases (Mandelshtam and Vasilyev, 2008; Defesche et al., 2017). Some mutations in the sterolin genes, *ABCG5/ABCG8*, which usually cause sitosterolemia, can also lead to FH in compound heterozygotes, while mutations in other genes, e.g., *APOE*, *STAP1*, and *LIPA*, are extremely rare in FH.

This review presents data on mutations identified in genes causing heterozygous FH in Russia. Polymorphisms in *LDLR* and *APOB* in the Russian population as a whole, but not in families with verified FH, are not discussed.

## Frequency of Familial Hypercholesterolemia in Russia

Familial hypercholesterolemia is the most frequent inherited disease in humans (Defesche et al., 2017). The frequency of heterozygous FH is estimated as 1 case per 500 examined individuals, while the homozygous form occurs in 1 in 1,000,000 (Goldstein et al., 2001); however, studies using standard diagnostic criteria have been conducted in a number of countries, revealing FH frequencies approximately twice as high in outbred populations (1:219–1:300). The corresponding figure for homozygous mutations was 1:300,000 (Defesche et al., 2017; Sarraju and Knowles, 2019). Frequencies can be much higher in populations with evident founder effects.

No attempts were made to evaluate FH frequency in Russia for many years. Present estimates show that the number of patients heterozygous for mutations causing FH among the approximately 144.5 million population of Russia varies between 287,000 (Karpov et al., 2015) and 1,300,000 (Ershova et al., 2017), while approximately 150–300 people have the homozygous form of FH (Karpov et al., 2015). This wide range of values results from differences in the criteria used to diagnose FH and in patient selection criteria. In some studies the number of patients with a family history of hypercholesterolemia were calculated, while other protocols considered the number of individuals with high cholesterol levels within the whole population screened. This reflects the absence of a consensus on the meaning of the term “familial hypercholesterolemia” (Masana et al., 2019).

Almost every estimate of FH frequency has been based on clinical, rather than genetic, criteria for FH diagnosis. A population study in West Siberia, conducted under the auspices of The Epidemiology of Cardiovascular Risk Factors and Diseases in Regions of the Russian Federation Study (ESSE-RF), is a very recent example of evaluation of the frequency of heterozygous FH in Russia (Ershova et al., 2017). That study included 1,630 and 1,622 individuals from the Tyumen and Kemerovo regions, respectively, with an age range of 24–65 years. The overall frequency of definite FH, diagnosed using the criteria of the Dutch Lipid Clinic Network (DLCN), was 0.24% (1 per 407 persons examined), while that of probable FH was 0.68% (1 per 148 examined); hence the total frequency of patients with definite or probable FH was 0.92% (1 per 108 examined). In that study, 40% of patients with definite or probable FH had coronary artery disease (CAD), while only 23% of patients received statin therapy. The odds ratios for development of CAD and myocardial infarction in the overall group of patients with definite or probable FH were, 3.71 (95% confidence interval (CI): 1.58–8.72; *p* = 0.003) and 4.06 (95% CI: 0.89–18.55; *p* = 0.070), respectively, relative to patients unlikely to have FH. The authors concluded that the frequency of FH in Russia may be significantly higher than previously considered, and that only a small proportion of patients with FH receive a correct diagnosis and adequate treatment. The high occurrence of FH in Russia reported by Ershova et al. (2017) may arise from the sampling, i.e., the group of patients with probable FH might contain not only patients with the disease in the direct sense of the term, but include individuals with polygenic inheritance as well. Therefore it is hard to compare these data with reports of other authors cited by Defesche et al. (2017).

The high risk of coronary atherosclerosis among patients with FH and the modes of preventive treatment currently practiced highlight the need to develop genetic investigations to elucidate the specific molecular features of FH in Russia.

## Studies of FH Genetics in Russia

### The LDLR Gene Mutation Spectrum in Saint-Petersburg

Molecular genetic research into FH in Russia was launched in 1987 at the Institute of Experimental Medicine in Saint-Petersburg. The work began by constructing molecular probes to search for large-scale rearrangements in the *LDLR* gene and to analyze its restriction fragment length polymorphism (RFLP) haplotypes. To meet this aim, a series of plasmids, based on the pLDLR-3 vector containing the full-length cDNA encoding LDL receptor, were constructed (Yamamoto et al., 1984). Sub-cloning of *LDLR* fragments was required, since the 3′-untranslated region of the mRNA contained Alu repeats, which had to be eliminated to identify sequences unique to *LDLR*. Southern hybridization experiments using samples from 50 patients with verified clinical diagnoses of FH revealed a 5 kb deletion, encompassing exons 4–6 of *LDLR* segregating in a family with FH (Mandelshtam et al., 1993). These data demonstrate that large-scale rearrangements within *LDLR* are a rare cause of FH in Russia, while the majority of alterations are point mutations, which cannot be detected by Southern hybridization. These findings are consistent with current notions of the global distribution of mutations in *LDLR*; that is, that partial deletions comprise 8–10% of all genetic abnormalities identified at this locus (Bourbon et al., 2017; Defesche et al., 2017).

A second large deletion in *LDLR* was identified in the Russian population by geneticists in Moscow after the introduction of targeted sequencing (Averkova et al., 2018). As Southern blot hybridization is a time-consuming and relatively inefficient method for mutation screening, no more attempts to use this approach to identify large-scale rearrangements at the *LDLR* locus have been undertaken in Russia.

At the very beginning of the study of FH in Saint-Petersburg, it became clear that various *LDLR* RFLP haplotypes segregate with the clinical phenotype, which is indicative of the variety of mutations, the molecular heterogeneity of the disease, and the absence of profound founder effects (Mandel’shtam et al., 1995). Further research into the mutation spectrum at *LDLR* confirmed those findings (Zakharova et al., 2005, 2007). Early studies of FH genetics in Saint-Petersburg provided evidence of varying degrees of atherogenic manifestations within a separate family carrying the same genetic defect, leading to increased plasma cholesterol concentration. This observation implies the participation of other endogenous and exogenous factors in generating the biochemical and clinical manifestations associated with FH (Lipovetsky et al., 1996).

A new stage of the study of FH in Russia began with the introduction of PCR into routine practice. The first experiments with PCR in Saint-Petersburg were conducted in the Nuclear Physics Institute by E. I. Schwartz. Rapid dissemination of PCR soon allowed its use to study *LDLR*. The longest exon, exon 4 (Südhof et al., 1985), became the first target of searches for point mutations. Exon 4 was considered most likely to contain mutations, as it encodes the longest amino acid stretch in the functionally important ligand-binding domain of the LDLR protein. A set of primers designed by Hobbs et al. (1992) was used to search for mutations and mutant species were selected for manual sequencing by screening using single-strand conformation polymorphism (SSCP) analysis (Orita et al., 1989a,b), visualized by silver-staining of DNA in polyacrylamide gels (Bassam et al., 1991).

The first point mutations in *LDLR* identified in Saint-Petersburg were c.478T > G p.Cys160Gly (initially designated C139G) and c.444T > G p.Cys148Trp (initially termed as C127W) (Supplementary Table 1). Those alterations had never previously been described in other world populations, hence they were termed “Slavic” mutations (Chakir K. H. et al., 1998), although the ethnicity of the patients had not been verified, except that they reported themselves of Russian origin. Interestingly, p.Cys148Trp has never since been detected in other Russian families. In contrast, p.Cys160Gly has subsequently been detected several times; for example, in another family from Saint-Petersburg (unrelated to the first), and in probands from Novosibirsk (Mandelshtam and Maslennikov, 2001) and Moscow (Meshkov et al., 2004, 2009). Subsequently, it became clear that p.Cys160Gly is the most frequent mutation found among ethnic Russian populations. No other “Slavic” mutations were detected in four unrelated families (Supplementary Table 1). Later, collaborative research conducted by the Laboratory of Human Molecular Genetics, headed by E. I. Schwartz, and the Department of Molecular Genetics of the Institute of Experimental Medicine, headed by V. I. Gaitskhoki, identified several other mutations in *LDLR* using the same screening methods (Chakir K. et al., 1998; Mandelshtam et al., 1998; Zakharova et al., 2001; Tatishcheva et al., 2001; Supplementary Table 1).

The introduction of highly sensitive automated SSCP analysis in DNA gel analyzers (e.g., the “ALFExpress-2” from Amersham), as well as automated fluorescent DNA sequencing, stimulated further progress in the study of the molecular basis of FH in Saint-Petersburg. Consequently, systematic investigations documented 33 mutations in *LDLR*, 18 of which had never been described elsewhere in the world (Supplementary Table 1; Zakharova et al., 2005, 2007; Mandelshtam, 2011). These findings demonstrated the high variability of the *LDLR* gene and supported the virtually complete absence of a founder effect in the Saint-Petersburg population (Zakharova et al., 2005, 2007). Mutations were discovered in 59% (44 of 74) of probands in Saint-Petersburg with verified FH in whom all exons of *LDLR* were completely investigated, including alterations that (in our opinion) likely caused the disease in 55% of families with FH (41 of 74); however, the most interesting result was not finding novel and/or known mutations in families considered separately, which was expected in the poorly explored Russian population, but detection of mutations that were ethnically specific. Two mutations typical of neighboring Finland were found in Saint-Petersburg: a seven-nucleotide deletion, c.925-931del (FH North Karelia) and c.1202T > A p.Leu401His (FH Pori) (Zakharova et al., 2005). The variant widely detected in Eastern Finland, FH North Karelia (Koivisto et al., 1992), was detected in a Finnish family from Petrozavodsk (Komarova et al., 2013a,b, c). Use of allele-specific PCR did not allow detection of the 9.5 kb *LDLR* deletion prevalent in Finland, FH Helsinki c.2311 + 245_c.^∗^807del p.771-860delins55aa), in either the Saint-Petersburg population, or among patients with FH in Karelia (Komarova et al., 2013a).

Mutations typical of the Finnish population appeared to be rare, and were found in only a few families, which can be explained by the absence of ethnic Finns among the patient sample. The absence of FH Helsinki among Russians resident in Saint-Petersburg, alongside Finns, is not surprising, since miscegenation has been prevented by linguistic and religious differences, especially in the times of the Russian Empire; hence, Russians and Karelians practicing Orthodox religion seldom married Finnish Lutherans. Notably, the Finnish population differs significantly from those in neighboring countries, as mutation spectra associated with other hereditary diseases, such as phenylketonuria and familial breast cancer, among others, are commonly specific for that ethnic group. Hence, it is predictable that typically Finnish mutations in *LDLR* are rare in North-West Russia.

The situation among the Ashkenazi Jewish subpopulation in Saint-Petersburg appears very different. The common c.654_656del p.Gly219del LDLR pathogenic variant was detected (Mandelshtam et al., 1998) in 7 of 22 (32%) families with FH. This mutation had been previously described as the allele FH-Lithuania (Meiner et al., 1991) and is known mostly under its initial designation, G197del or c.652_654delGGT in the literature (Supplementary Table 1). This trinucleotide deletion was previously identified in 35% of Ashkenazi with FH in Israel, of which 64% (16 of 25) originated from Lithuania (Meiner et al., 1991). An even higher frequency (8 of 10 families) of FH-Lithuania was observed in the Jewish community of the South African Republic, which formed as a result of mass-scale immigration of Jews from Lithuania at the turn of the 20th century (Meiner et al., 1991).

The FH-Lithuania mutation was studied in 78 Ashkenazi probands from Israel, Russia, South Africa, the Netherlands, and the United States (Durst et al., 2001). All patients confirmed that their families originated from Lithuania and carried the mutation on a chromosome with a common haplotype. Analysis of flanking DNA markers allowed the mutation to be dated to the early 14th century, which coincides with the settling of Jews in Lithuania (1338 A.D.) and with the years of rapid growth of the Jewish population in Eastern Europe (Durst et al., 2001). The high frequency of p.Gly219del among Ashkenazi Jews has been proposed to be the result of a founder effect, rather than natural selection (Durst et al., 2001; Risch et al., 2003).

The Jewish community of Saint-Petersburg essentially consists of Ashkenazi and originates mostly from the western regions of the Russian Empire, Lithuania, and Poland (Mandelshtam et al., 1998). The FH-Lithuania mutation has been found in 14 patients from Saint-Petersburg and in an Ashkenazi proband from Novosibirsk (Mandelshtam and Maslennikov, 2001). Other than individuals with Ashkenazi origin, the same mutation was found in two Saint-Petersburg families with no record of Jewish origin (described as c.651_653delTGG in Zakharova et al., 2005).

Patients homozygous for p.Gly219del are characterized by low residual LDL receptor activity (approximately 2%) (Hobbs et al., 1992), termed the receptor-negative phenotype. Biochemical analysis of patient samples from Saint-Petersburg clearly demonstrated that lipid profile aberrations are more pronounced in patients heterozygous for the “Lithuanian” mutation than in the group comprising other patients carrying various non-characterized mutations (Lipovetsky et al., 1998). Patients with FH-Lithuania also had more severe symptoms related to FH than those in the combined group. Further, they were resistant to treatment with fluvastatin (Lipovetsky et al., 1998). The FH-Lithuania mutation has not been found in Karelia to date (Komarova et al., 2013c; Korneva et al., 2017a) or in Moscow (Meshkov et al., 2009).

### The LDLR Mutation Spectrum in Patients From Moscow With FH

Molecular genetic studies of FH in Moscow began in 2001 (Krapivner et al., 2001). Unlike researchers in Saint-Petersburg, their colleagues in Moscow chose a better set of primers to amplify the *LDLR* gene (Jensen et al., 1996), which permitted analysis of all the exon-intron boundaries, rather than only the sequences of exons and some of the invariant splicing sites detected by the primers introduced by Hobbs et al. (1992), which were used in Saint-Petersburg and Petrozavodsk.

A two-step protocol was applied to detect mutations, comprising exon screening by SSCP, where conformers were stained using ethidium bromide, fluorescent dye (Krapivner et al., 2001), or SYBR Gold (Meshkov et al., 2004, 2009), and automatic sequencing of selected samples. This approach was used to conduct molecular analysis of *LDLR* in 50 patients with heterozygous FH, facilitating detection of 21 potentially disease-causing mutations in 23 patients (46%), excluding several frequent polymorphisms. Only two mutations (c.1246C > T p.Arg416Trp and c.2389 + 5G > A) were detected in two families, with all of the other 19 mutations each present in a single family.

The mutation spectrum of *LDLR* in the Moscow cohort was rather peculiar. The published results indicated that 15 of 21 mutations (71%) were specific to Russia, with only 6 (29%) previously reported in other countries (Meshkov et al., 2009). By 2020 the spectrum of mutations in *LDLR* had extended, and our analysis shows that only 9 (43%) of all mutations previously described (Meshkov et al., 2009) remain specific for Russia, while the remaining 12 (57%) are shared with other populations. This conclusion is supported by analysis of previous data obtained from the Saint-Petersburg population (Zakharova et al., 2005, 2007; Mandelshtam, 2011). Indeed, calculations made in 2020 show that only 12, rather than 18, of 33 mutations studied (36%) are specific to Russia, while 21 of 33 mutations (64%) have been described elsewhere.

The results obtained in the two largest Russian cities provide evidence that some 40% of mutations in *LDLR* are specific to Russia. Only two mutations (p.Cys160Gly and c.810G > A p.Cys270Ter) were common among the populations of Moscow and Saint-Petersburg (Zakharova et al., 2007; Meshkov et al., 2009). At present, DNA-based diagnosis of *LDLR* mutations in both Moscow and Saint-Petersburg is conducted by routine commercial analysis; therefore, no summary data on FH mutations are published.

New expectations of more efficient DNA-based diagnosis of FH have been raised by the introduction of targeted sequencing in routine practice. A recent study of 38 patients from Moscow with clinically diagnosed FH and acute coronary syndrome revealed genetic abnormalities in 24 individuals (63.2%) (Averkova et al., 2018); however, mutations in *LDLR* were found in only 10% of probands (four sequence variants, of which one (c.58G > A p.Gly20Arg) was experimentally demonstrated to be neutral). This result is at variance with data from previous studies conducted in Russia and elsewhere in the world, showing that patients with *LDLR* mutations constitute the great majority (usually 70–80%) of cases with a hereditary background (Defesche et al., 2017). This discrepancy likely arose from the distinctive method of sampling and inaccuracy of the clinical FH diagnosis, making the results obtained (Averkova et al., 2018) incomparable with earlier data (Meshkov et al., 2009).

When this review was ready for submission a report was published on a cohort of 52 probands with definite or probable diagnosis of FH according to DLCN criteria (Semenova et al., 2020). Targeted NGS was used to study that cohort consisting of individuals from various regions of Russia who had settled in Moscow. Genetic defects were revealed in 48% of index cases i.e., 24 probands with mutations in the *LDLR* gene and 2 with mutations in the *APOB* gene were identified. 22 mutations in the *LDLR* gene were identified as pathogenic or likely pathogenic. Of those six mutations (but not eight as stated by the authors) were first described. Six mutations (but not three as the authors wrote) had been found in Russia previously. That study allowed to indicate 16 novel *LDLR* mutations in Russia. The most common pathogenic variant in *LDLR* was c.1775G > A p.Gly571Glu found in four probands. It had been previously reported as a repeatedly occurring mutation in St. Petersburg (Zakharova et al., 2001, 2007) and in Novosibirsk (Voevoda et al., 2008), also found in a number of populations in the world. Despite using NGS the percentage of probands with pathogenic variants was low, which probably results from the inaccuracy of the clinical FH diagnosis.

### LDLR Mutation Spectrum in Patients With FH From Petrozavodsk

In Petrozavodsk, the molecular basis of FH was studied using a similar approach to that applied in Saint-Petersburg. DNA was collected from 102 patients with definite FH, including 94 probands, according to DLCN criteria (Defesche et al., 2017). The presence of the c.10580G > A p.Arg3527Gln (R3500Q) mutation in *APOB*, which generates non-binding apolipoprotein B-100 (FDB, familial-defective apoB-100) was ruled out in all samples, using allele-specific PCR (Hansen et al., 1991). Patients with FH were also tested for the two major Finnish mutations: the 9.5 kb deletion FH Helsinki (Aalto-Setälä et al., 1988; Aalto-Setälä, 1988) and the 7-nucleotide deletion c.925_931delCCCATCA (known as FH-North Karelia) (Koivisto et al., 1992). The first of these two mutations was detected by allele-specific PCR (Kontula et al., 1992), while for the second heteroduplex analysis and detection of single-strand conformers were conducted in *LDLR* exon 6 amplified by PCR (Komarova et al., 2013a). The FH Helsinki mutation was not found, while FH-North Karelia was detected in a single proband among 80, who reported he was of Finnish origin (*ibid*.). Subsequently, Cy-5 tagged primers (Hobbs et al., 1992; Jensen et al., 1996) were used to amplify separate exons, and samples with abnormal mobility were automatically sequenced (Komarova et al., 2013b,c). This analysis resulted in discovery of 14 different mutations capable of causing a clinical phenotype and a number of frequent polymorphisms in the cohort of 52 patients with completely sequenced *LDLR* genes (Supplementary Tables 1, 2; Komarova et al., 2013b,c; Korneva et al., 2013, 2014, 2016, 2017a,b).

Of the 52 patients subjected to genetic analysis, 22 probands (42%) carried mutations. Mutations likely to cause the disease were discovered in 14 families (27%) (Korneva et al., 2017a). Only the p.Leu646Ile mutation was found in two probands (Komarova et al., 2013b,c), while all other variants were detected in single families with FH, but not in controls (Komarova et al., 2013c; Korneva et al., 2013).

Interestingly, the spectrum of *LDLR* mutations in Karelia had few similarities with that found in Saint-Petersburg; the one common variant was FH-North Karelia (Komarova et al., 2013a,b, c). Further, the c.1327T > C p.Trp443Arg mutation was present in both the Karelian and Moscow populations (Korneva et al., 2017a). Of 14 mutations reported in the latter publication only 4 had been found elsewhere in the world, while the remaining 10 (71%) were specific for Russia (*ibid*.). The small size of the cohort studied may partly account for these differences. In our opinion, these results highlight the genetic peculiarity of the Russian population, with respect to the spectrum of *LDLR* gene mutations found in patients with FH (Komarova et al., 2013b; Korneva et al., 2017a).

### LDLR Mutation Spectrum in Patients With FH in Novosibirsk

Studies of FH accomplished in Novosibirsk to date were conducted in three stages. First, patients were screened for mutations discovered in Saint-Petersburg (Mandelshtam and Maslennikov, 2001) and families carrying p.Cys160Gly and p.Gly219del identified. Second, *LDLR* coding regions were sequenced in samples from 20 patients aged 45–49 years with the highest levels of serum cholesterol, disregarding family history of hypercholesterolemia (Voevoda et al., 2008). This approach facilitated detection of 7 novel and 12 previously described mutations in *LDLR* (Supplementary Table 1), however, 4 of the 7 novel mutations identified were silent, and one variant appeared to be a well-known neutral polymorphism. The remaining variants were not tested functionally and no *in silico* predictions were conducted. Further, as there was no familial analysis, the importance of the newly detected mutations for the etiology of hypercholesterolemia remains unclear. Nevertheless, this research was meaningful, since it revealed a significant difference between the *LDLR* mutation spectrum in a population sample of patients with hypercholesterolemia and that among patients with FH (Voevoda et al., 2008).

In Novosibirsk, mutation screening of patients with hypercholesterolemia is currently conducted by targeted sequencing of several genes involved in the development of monogenic FH (i.e., *LDLR*, *APOB*, *PCSK9*, and *LDLRAP1*). The most recent results demonstrate the efficiency of this approach (Shakhtshneider E. et al., 2019; Shakhtshneider E. V. et al., 2019), which facilitated examination of 42 patients with clinically diagnosed definite FH, resulting in detection of 12 different mutations in *LDLR* in 17 probands and two cases with a p.Arg3527Gln mutation in *APOB*. Thus, mutations were identified in 45% of families, of which 40% had changes in *LDLR* and 4.8% in *APOB*. No mutations associated with FH were found in *PCSK9*. Similarly, no homozygotes or compound-heterozygotes for mutations in *LDLRAP1* (ARH) were observed. Of the 12 mutations in *LDLR*, 10 were missense, one was localized in a splice donor site (Shakhtshneider et al., 2017a), and the other was a deletion, however, the mutations were not detailed in the abstract (Shakhtshneider E. V. et al., 2019).

### Why Is It Challenging to Compare Research Carried out in Different Regions of Russia?

As described above, there have been a limited number of investigations into the mutation spectrum of FH in Russia. The studies that have been conducted differed noticeably in design, making comparisons among them difficult. The best characterized, with respect to the *LDLR* mutation spectrum, are the populations of Saint-Petersburg, Moscow, Petrozavodsk, and Novosibirsk. The clearest differences among those studies are as follows: (1) different methods of study cohort selection; (2) different primer sets; and (3) incomparable methods of mutation screening. Below, we consider each of these dissimilarities separately.

#### Differences in Patient Selection Criteria

In both Saint-Petersburg and Moscow, patients were primarily selected based on familial history of disease, whereas researchers in Novosibirsk used population sampling of individuals aged 45–49 years with high levels of serum cholesterol (Voevoda et al., 2008). Further, the following criteria were used for FH clinical diagnosis in Saint-Petersburg (Mandelshtam et al., 1993): (1) type IIa or type IIb hyperlipidemia, featuring high cholesterol, particularly LDL cholesterol; (2) several members of the proband’s family have hypercholesterolemia or reported myocardial infarctions at an early age; (3) the patient has tendinous xanthomas and/or lipoid corneal arches. Only patients meeting at least two of these criteria were included in the study. In Petrozavodsk, patient selection and diagnosis of FH were largely based on the recommendations of the DLCN (Korneva et al., 2017a), while researchers in Moscow relied on MEDPED (The Make Early Diagnosis To Prevent Early Death) FH program or Simon Broom Register criteria (Meshkov et al., 2009).

#### Different Sets of Primers Used to Search for *LDLR* Gene Mutations

Different sets of primers were used to amplify *LDLR* gene exons and were not detailed in scientific publications. Only the coding region of the gene was studied in Novosibirsk by direct sequencing (Voevoda et al., 2008); hence, mutations in introns were not detected. The set of primers suggested by H. Hobbs et al. (1992) was used in Saint-Petersburg and Petrozavodsk to amplify the majority of *LDLR* amplicons. These primers facilitated full analysis of the gene exon sequences, however, only 12 of 34 exon-intron boundaries, which contain signals crucial for splicing, were covered by this primer set. An alternative pair of primers was used to amplify *LDLR* exon 3 alone (Jensen et al., 1996), facilitating detection of novel mutations in the splice donor site of intron 3, including the c.313 + 1G > A transition in two probands among 74 from Saint-Petersburg and a c.313 + 2G > T transversion in one proband of 52 from Petrozavodsk. Nevertheless, even with sequencing of the intron 3 boundaries, more than half the *LDLR* gene exon-intron interfaces were not covered in either the Saint-Petersburg or Petrozavodsk investigations. Researchers in Moscow used a more advantageous set of primers (Jensen et al., 1996; Meshkov et al., 2004, 2009), which allowed exploration of all exon-intron interfaces.

Notably, at least one study with an optimized *LDLR* mutation search strategy showed that up to 27% of patients can carry mutations that potentially disturb splicing; of 54 mutations identified by the authors, 13 were found in introns (Amsellem et al., 2002). That work is of great interest, although the contribution of intron mutations to the development of FH suggested by the authors should be treated with caution. Studies reported in this review usually did not explore *LDLR* introns deeply and so far only the results relating to the *LDLR* coding region can be directly compared in the various studies conducted in Russia.

#### Methods for Mutation Screening and Identification

The majority of methods used to look for mutations in *LDLR* in the Russian population aimed to identify point mutations, and short deletions and insertions, which can be detected by PCR and direct DNA sequencing. Less attention has been paid to searching for large-scale rearrangements at the *LDLR* locus. To date, such research in Russia has been limited to identification of two deletions. The first is a single 5 kb deletion that eliminates exons 4–6 of *LDLR*, found in two individuals of the same descent among 50 patients studied in Saint-Petersburg by Southern hybridization (Mandelshtam et al., 1993). The other is a deletion of 1,468 bp revealed by targeted sequencing of *LDLR* in Moscow (Averkova et al., 2018). Initial studies showed that large-scale rearrangements within the *LDLR* locus seldom cause FH in the population of Saint-Petersburg (Mandelshtam et al., 2004). Subsequently, research in Russia focused on other issues, despite the appearance of novel methods for identification of deletions and exon duplications; for example, real-time PCR.

In ethnic isolates, such as French-speaking Canadians, deletions can constitute more than 50% of all mutations causing FH (e.g., the French Canadian-1 and French Canadian-5 mutations) (Hobbs et al., 1992). Meanwhile in ethnically heterogeneous populations, for example, English-speaking Canadians, large deletions are found in 5–7% cases (Langlois et al., 1988). Since analysis of large-scale rearrangements of the *LDLR* locus has only been conducted in two Russian studies, one cannot compare different regions and various ethnic groups in this country with respect to the frequency of such mutations.

Various methods of searching for mutations at the *LDLR* locus have been used in different studies. Most involved amplification of separate exons and adjacent introns, as well as exon screening by DNA conformational polymorphism analysis, but not direct sequencing of exons. To identify single-strand conformers, silver staining of gels was used following electrophoresis (Chakir K. H. et al., 1998; Chakir K. et al., 1998; Mandelshtam et al., 1998; Tatishcheva et al., 2001; Zakharova et al., 2001). Alternatively, DNA was stained in gels using the fluorescent dye, SYBR Gold (“Molecular Probes”) (Krapivner et al., 2001; Meshkov et al., 2004, 2009). SSCP analysis was conducted using polyacrylamide, rather than mutation detection enhancement (MDE) gels. Analyses were often conducted at a single maintained temperature; for example, at 4°C (Meshkov et al., 2004) or 20°C (Tatishcheva et al., 2001; Zakharova et al., 2001). Some protocols did not require the addition of 10% glycerol to the polyacrylamide gels, diminishing the efficiency of SSCP analysis. Hence, even the most extensive research accomplished during the previous decade in Moscow, only detected mutations in 23 of 50 (46%) probands with FH (Meshkov et al., 2009). An efficiency of SSCP analysis for mutation screening comparable with direct sequencing can be achieved only when varied conditions and MDE gels are used (Jensen et al., 1996).

Automated fluorescent SSCP analysis noticeably increased the efficiency of the method, making it comparable with direct automated DNA sequencing. In our study (Zakharova et al., 2005) fluorescent SSCP analysis was used to reveal *LDLR* mutations in 56% (14 of 26) of patients, while direct sequencing of all exons allowed detection of those in 53% (10 of 19) of probands. Our results indicate the high efficiency of fluorescent SSCP analysis in screening for *LDLR* mutations. Subsequently, this approach was comprehensively applied in studies conducted in Petrozavodsk (Komarova et al., 2013c; Korneva et al., 2017a).

### Mutations in the APOB Gene and Their Contribution to FH Etiology

Frequent mutations in exon 26 of *APOB* in a large sample of 730 patients from Moscow with FH (OMIM #143890) indicated that patients with such changes comprise approximately 2.6% of cases (Meshkov et al., 2009). Among all variants found in Moscow, only the p.Arg3527Gln (R3500Q) mutation is unambiguously associated with hypercholesterolemia, as it causes a deficiency of binding with the ApoB-100 receptor (FDB); it was found in 14 (1.9%) patients with FH (Meshkov et al., 2009).

A smaller scale study, including 111 patients with the heterozygous form of FH, identified five heterozygotes (4.5%) (Malyshev et al., 2007), with at least three families where patients carried p.Arg3527Gln originating from Central Russia. In four families, the carriers were ethnic Russians, and in one family the mutation was supposedly inherited from the Lithuanian mother (*ibid.*). This is an indication that p.Arg3527Gln, which is most frequent in Switzerland, Austria, and Germany (Hansen, 1998), is also disseminated in Eastern Europe. The p.Arg3527Gln mutation was found in only 2 of 42 patients (4.8%) on high throughput targeted sequencing of *APOB* in patients with verified FH in Novosibirsk (Shakhtshneider E. V. et al., 2019).

Allele-specific PCR, used in a purposeful search for R3500Q mutation in *APOB* among 74 probands with FH from Saint-Petersburg and 52 probands from Karelia, generated negative results (Komarova et al., 2013b,c; Korneva et al., 2017a). In comparison, p.Arg3527Gln was found in 6.6% of independently reported cases (25 of 378) of autosomal dominant hypercholesterolemia in Poland (Chmara et al., 2010). No clear link has been proven between other *APOB* mutations found in Moscow and FH development.

### Mutations in PCSK9 and Their Contribution to FH Etiology

Globally, the contribution of *PCSK9* gene mutations does not exceed 1% of all FH cases (Defesche et al., 2017). Only gain-of-function mutations, which intensify receptor degradation, cause the autosomal-dominant form of FH, while loss-of-function mutations favor a decrease in cholesterol levels (Abifadel et al., 2009). A vivid example of the low frequency of such mutations is provided by a study of a French FH patient cohort, involving 1,358 probands from various regions (Marduel et al., 2010), where the authors found mutations in *PCSK9* in only 0.7% of probands.

Despite the rarity of *PCSK9* mutations, studies of their frequency were undertaken using targeted sequencing in Novosibirsk (Shakhtshneider et al., 2017b; Shakhtshneider E. et al., 2019; Shakhtshneider E. V. et al., 2019) and Moscow (Averkova et al., 2018). Different variants of the gene were found, which either favored hypocholesterolemia (c.137G > A p.Arg46Leu, rs11591147), or were of unclear function (Shakhtshneider et al., 2017b). To date, no single *PCSK9* variant capable of provoking FH has been detected in Russia (Shakhtshneider E. V. et al., 2019). The c.2009G > A p.Gly670Glu (rs505151) variant was regarded as possibly favoring FH development (Abifadel et al., 2009); however, in the Russian population this variant is found at equal frequencies among individuals with hyper- and normo-cholesterolemia (Astrakhova et al., 2016) and, therefore, cannot be considered a cause of FH. Concerning other variants of *PCSK9* discovered in Russian populations, no comparisons were made between patients with FH and the control group; hence, identification of *PCSK9* mutations associated with FH in Russia has yet to occur.

## Discussion

The percentage of FH patients with identified mutations causing the clinical phenotype in various regions of Russia is lower than that among other world populations; there are several reasons for this situation. First, the majority of studies in Russia were dedicated to searching for mutations in the *LDLR* gene, but not *APOB*, which can be responsible for approximately 10–15% of FH cases (Defesche et al., 2017). Second, screening for large-scale rearrangements in *LDLR* has not been comprehensive, and this type of alteration can cause 8–10% of FH cases in populations with no clear founder effect. Third, a number of studies focused primarily on the *LDLR* coding region, with no analysis of exon-intron boundaries or the promotor region (Voevoda et al., 2008). Finally, and probably the most important the patient samples included not only patients with definite FH, but also those with probable FH, as judged by the DLCN criteria (Defesche et al., 2017) that may be caused not by monogenic mutations.

The actual percentage of families with identified *LDLR* mutations in Russia may be even lower than reported in the literature cited, since the samples of patients with FH subjected to genetic testing were relatively small, not exceeding 100 probands. Genetic analysis of 50 patients with clinical FH revealed 21 causative mutations in 23 individuals (46%) (Meshkov et al., 2009). Overall, the percentage of *LDLR* mutations among 74 probands in Saint-Petersburg was 59% (44 of 74), of which 55% were causative (41 of 74) (Zakharova et al., 2005; Mandelshtam, 2011). Of 52 patients with clinical FH studied in Karelia, 22 probands (42%) carried pathogenic mutations in *LDLR* (Komarova et al., 2013c; Korneva et al., 2017a). Targeted sequencing of *LDLR*, *APOB*, *PCSK9*, and *LDLRAP1* in 42 patients with verified FH from Novosibirsk revealed nucleotide substitutions in 45% of patients that would be expected to cause inadequate receptor function (Shakhtshneider E. et al., 2019; Shakhtshneider E. V. et al., 2019). In comparison, in the Netherlands, screening of *LDLR* and *APOB* in patients with an FH clinical phenotype revealed causative mutations in 77% of patients (Fouchier et al., 2005). High-resolution melting analysis allowed detection of such mutations in 17 of 28 (61%) patients with FH of Greek origin (Whittall et al., 2010). Further, a Turkish study revealed mutations causing FH in 37 of 56 (66%) alleles tested (Sözen et al., 2005), while a genetic study of FH in a French population detected mutations in 73.9% of patients with autosomal-dominant hypercholesterolemia (Marduel et al., 2010). Moreover, 6.6% of patients carried *APOB* mutations, and 0.7% had mutations of *PCSK9*. In only 19% of patients, no mutations associated with FH were detected (*ibid*.). An investigation of a large cohort of 1,018 Italian patients with autosomal-dominant hypercholesterolemia revealed mutations in 832 probands (82%), of whom 97.4% had mutations in *LDLR*, 2.2% in *APOB*, and 0.36% in *PCSK9* (Bertolini et al., 2013). A cohort of 378 patients with FH was studied in Poland, of which 234 (62%) carried variants of *LDLR* and *APOB*; the researchers found 99 *LDLR* sequence variants and one mutation (p.Arg3527Gln; R3500Q) in *APOB* (Chmara et al., 2010). Of the 99 *LDLR* variants, 71 were potentially pathogenic mutations (*ibid.*).

In Russia the majority of *LDLR* mutations were discovered in unique families. This can be attributed to the relatively small sample sizes of patients with FH. At present, the peculiarity of the *LDLR* mutation spectrum in Russia cannot be precisely evaluated.

Rapid improvements in next generation sequencing and the increasing number of sequenced genomes are likely to show that genetic variations previously regarded as specific for certain countries are also present in different populations around the world. Data presented in Supplementary Material allow calculation of a rough estimate of the peculiarity of the *LDLR* mutation spectrum in Russia. Some calculations were made after excluding silent mutations and neutral polymorphisms, which are more widespread than mutations causing FH.

As mentioned above, approximately 40% of the mixed populations of Moscow and Saint-Petersburg present with mutations specific to Russia. Despite the lower number of families subjected to DNA-testing in Karelia, only 4 of 14 *LDLR* mutations capable of causing FH detected in this region have been described elsewhere in the world, while the other 10 (71%) were reported as novel (Korneva et al., 2017a). Karelian data seem more typical of the Russian population as a whole, relative to those obtained in Moscow, Saint-Petersburg, and Novosibirsk. In the latter megapolis, only 1 of 10 mutations causing FH had not been described in other parts of the world (Shakhtshneider E. et al., 2019). Therefore, to identify “genuine” Russian mutations associated with the development of FH, future research should be conducted in rural settlements, or small towns with majority ethnic Russian populations. To date, collected samples of genetically characterized patients in Russia have been small, and it is difficult to specify major mutations that could reflect national features of FH, particularly as the majority of studies cited in this review did not explore possible associations of specific mutations with the ethnic origin of patients (Zakharova et al., 2005; Voevoda et al., 2008; Meshkov et al., 2009; Komarova et al., 2013c; Korneva et al., 2017a; Shakhtshneider E. et al., 2019; Shakhtshneider E. V. et al., 2019). It seems likely that major achievements in the genetic investigation of the molecular features of FH in Russia are yet to come.

## Author Contributions

FZ and TB elaborated data, composed tables, and edited the text. MM and VV wrote and composed the text. All authors contributed to the article and approved the submitted version.

## Conflict of Interest

The authors declare that the research was conducted in the absence of any commercial or financial relationships that could be construed as a potential conflict of interest.
