# Supplementary material for: Familial Hypercholesterolemia in Russia: Three Decades of Genetic Studies
Source: Front Genet. 2020 Dec 17;11:550591. doi: 10.3389/fgene.2020.550591 (PMC7773754; doi:10.3389/fgene.2020.550591)
Supplement: Supplementary file 1 [file Table_1.docx]

**Supplementary Table 1. Mutations detected in the *LDLR* gene in Russia**

| Exon No. | Nucleotide substitution  NM_000527.4  (LDLR) | HGVS nomenclature NG_009060.1  (LDLR)  (LRG 274) | Genomic location GRCh38 (Chr19) p12 | Predicted effect on protein | HGMD Public Accession | No. of families | ClinVar estimate of pathogenicity, Accession | dbSNP rs number | Populations in Russia | References for Russia | Other populations in the world |
| --- | --- | --- | --- | --- | --- | --- | --- | --- | --- | --- | --- |
| **Nonsense mutations** | | | | | | | | | | | |
| 2 | c.85A>T  AGA>TGA Originally erroneously described c.85T>A | g.15860A>T | 11100240 | p.R29*  [p.Arg29Ter, R8X]  Originally erroneously described as E8X | CM045798  Disease causing mutation | 1 | Pathogenic  Accession: VCV 000251011.1 | [rs879254401](https://www.ncbi.nlm.nih.gov/snp/rs879254401) | Moscow | Meshkov et al., 2004, 2009 | None |
| 2 | с.97C>T  CAG>TAG | g.15872C>T | 11100252 | p.Q33*  [p.Gln33Ter Q12X, FH Turkey] | CM920404  Disease causing mutation | 1 | Pathogenic  Accession: VCV 000003683.4 | [rs121908024](https://www.ncbi.nlm.nih.gov/snp/rs121908024) | St. Petersburg | Zakharova et al., 2005, 2007 | France, Italy, Spain, Turkey |
| 3 | c.285C>A  TGC>TGA | g.18378C>A | 11102758 | p.C95*  [p.Cys95Ter, C74X] | CM983442  Disease causing mutation | 1 | Pathogenic  Accession: VCV 000251115.1 | [rs139400379](https://www.ncbi.nlm.nih.gov/snp/rs139400379) | St. Petersburg | Zakharova et al., 2001, 2007 | Japan, Korea |
| 4 | c.495G>A  TGG>TGA | g.21021G>A | 11105401 | p.W165*  [p.Trp165Ter, W144X] | CM067692  Disease causing mutation | 1 | Pathogenic.  Accession: VCV 000251254.1 | [rs879254546](https://www.ncbi.nlm.nih.gov/snp/rs879254546) | Moscow | Meshkov et al., 2009 | China, Greece |
| 4 | c.639G>A  TGC>TGA | g.21219C>A | 11105599 | p.C231*  [p.Cys231Ter, C210X] | CM930475  Disease causing mutation | 1 | Pathogenic/ Likely pathogenic. Accession: VCV 000003730.4 | [rs121908035](https://www.ncbi.nlm.nih.gov/snp/rs121908035) | Moscow | Meshkov et al., 2009 | Australia, Czech Republic, Great Britain, French Canadian, Ireland, Northern Poland, etc. |
| 4 | c.682G>T  GAG>TAG | g.21208G>T | 11105588 | p.E228*  [p.Glu228Ter, E207X, FH Morocco] | CM920425  Disease causing mutation | 1 | Pathogenic /Likely pathogenic  Accession:  VCV 000226333.8 | [rs121908029](https://www.ncbi.nlm.nih.gov/snp/rs121908029) | St. Petersburg | Zakharova et al., 2005, 2007 | China, Czech Republic, Germany, Great Britain, Korea, Morocco, The Netherlands,  Norway, Sweden, USA |
| 5 | c.718G>T  GAA>TAA | g.22208G>T | 11106588 | p.E240* [p.Glu240Ter] | CM055368  Disease causing mutation | N/A | Pathogenic  Accession: VCV 000251422.1 | [rs768563000](https://www.ncbi.nlm.nih.gov/snp/rs768563000) | Novosibirsk | Voevoda et al., 2008 | The Netherlands, USA |
| 5 | c.810G>A  TGC>TGA | g.22300G>A | 11106680 | p.C270*  [p.Cys270Ter, C249X] | CM051098  Disease causing mutation | 2 | Pathogenic  Accession: VCV 000251465.1 | [rs773328511](https://www.ncbi.nlm.nih.gov/snp/rs773328511) | St. Petersburg, Moscow | Zakharova et al., 2005, 2007; Meshkov et al., 2009 | The Netherlands |
| 9 | с.1252G>T  GAG>TAG | g.28963G>T | 11113343 | p.E418*  [p.Glu418Ter, E418X, E397X,  FH St Petersburg] | CM983997  Disease causing mutation | 1 | Pathogenic  Accession:  VCV 000251755.1 | [rs869320651](https://www.ncbi.nlm.nih.gov/snp/rs869320651) | St. Petersburg | Chakir et al., 1998 | Northern Italy |
| 9 | с.1328G>A  TAG>TGG | g.29039G>A | 11113419 | p.W443*  [p.Trp443Ter, W422X] | CM051099  Disease causing mutation | 2 | Pathogenic  VCV 000251789.1 | [rs879254866](https://www.ncbi.nlm.nih.gov/snp/rs879254866) | St. Petersburg | Zakharova et al., 2005, 2007 | Poland |
| 12 | c.1731G>A  TGG>TGA | g.32504G>A | 11116884 | p.W577*  [p.Trp577Ter, W556X, FH Moscow] | CM920462  Disease causing mutation | 1 | Pathogenic  Accession: VCV 000226370.1 | [rs875989928](https://www.ncbi.nlm.nih.gov/snp/rs875989928) | Moscow | Hobbs et al., 1992 | France, China |
| 15 | c.2215C>T | g.38868C>T | 11123248 | p.Q739*  [p.Gln739Ter] | CM024745 Disease causing mutation | 1 | Likely pathogenic/Pathogenic  Accession:  VCV000252258.5 | N/A | Moscow | Semenova et al., 2020 | Italy, Mexico, Japan, Taiwan |
| **Frameshift mutations** | | | | | | | | | | | |
| 3 | c.193_201 delins  GACTTCA | g.18286_18294delins GACTTCA | 11102666-11102674 | p.Ser65fs  [p.Ser65Asp fs*64, FsS44:D108X] | CX138996  Disease causing mutation | 1 | Pathogenic  Accession:  VCV 000251072.1 | [rs879254434](https://www.ncbi.nlm.nih.gov/snp/rs879254434) | Petrozavodsk | Komarova et al., 2013b,c; Korneva et al., 2013 | None |
| 3 | c.195dupT  [c.195_196insT] | g.18288dup | 11102668 | p.Val66fs  [p.Val66Cys  fs*64),  FsV45: D108X] | CI136948 Disease causing mutation | 1 | Pathogenic  Accession:  VCV 000251075.1 | [rs879254435](https://www.ncbi.nlm.nih.gov/snp/rs879254435) | Petrozavodsk | Komarova  et al., 2013b,c | None |
| 3 | c.230dupG [c.230_231insG] | g.18323dup | 11102703 | p.Arg78fs  [p.Arg78Pro fs*52, G56fsX53] | CI045722,  Disease causing mutation | 1 | N/A | [rs879254440](https://www.ncbi.nlm.nih.gov/snp/rs879254440) | Moscow | Meshkov et al., 2004, 2009 | None |
| 4 | с.351_352 insTTCC  Originally described as c.352_355 insTTCC | g.20877_20878 insTTCC | 11105258 | p.Asp118fs  [p.Asp118Phe fs*13, D97FfsX13] | CI099861  Disease causing mutation | 1 | N/A | N/A | Moscow | Meshkov et al., 2009 | None |
| 4 | c.355_356insTTCC | g.20881_20882ins | 11105261_11105261 | p.(Gly119Valfs*)  [p.(Gly119Valfs*12)] | N/A  Likely pathogenic | 1 | N/A | N/A | Moscow | Semenova et al., 2020 | None |
| 4 | c.670dupG | g.21196dup | 11105575 | p.(Asp224fs)  [p.(Asp224Glyfs*4),  FsK202: S206X] | CI051351  Disease causing mutation | 1 | Pathogenic  Accession: VCV 000251372.1 | [rs879254629](https://www.ncbi.nlm.nih.gov/snp/rs879254629) | St. Petersburg | Zakharova et al., 2005, 2007 | None |
| 6 | [c.925_931del](https://www.ncbi.nlm.nih.gov/projects/sviewer/?id=NM_000527.4&search=NM_000527.4:c.925_931del&v=1:100&content=5)CCCATCA | g.23119_23125del | 11107499-11107505 | p.(Pro309fs)  [p.(Pro309Lysfs*59,  FsE287:V348X, FH North Karelia] | CD920880  Disease causing mutation | 2 | Pathogenic  Accession:  VCV 000003729.3 | [rs387906304](https://www.ncbi.nlm.nih.gov/snp/rs387906304) | St. Petersburg, Petrozavodsk | Zakharova et al., 2005, 2007; Komarova et al., 2013a,b,c | Finland, Norway, Sweden, USA. Typical for North Karelia in Finland |
| 6 | [c.939_940+3del](https://www.ncbi.nlm.nih.gov/nuccore/NM_000527.4?report=graph&mk=939\|NM_000527.4\\:c.939_940+3del\|green)  [c.936-940del5] | g.23133_23137del | 11107510-11107514 | p.(Glu291fs*)  [ FsE291: N309X] | CD051317  Disease causing mutation | 1 | Pathogenic  Accession: VCV 000251536.1  Considered as variant affecting splicing | [rs879254727](https://www.ncbi.nlm.nih.gov/snp/rs879254727) | St. Petersburg | Zakharova et al., 2005, 2007 | None |
| 9 | [c.1291_1331 del](https://www.ncbi.nlm.nih.gov/nuccore/NM_000527.4?report=graph&mk=1291\|NM_000527.4\:c.1291_1331del\|green)41 | g.29002_29042del | 11113377-11113422 | p.(Ala431fs)  [p.Ala431Ter, p.(Val430_Ala431insTer), FsV409:S423X] | CG052715  Disease causing mutation | 1 | Pathogenic  Accession: VCV 000251769.1 | [rs879254854](https://www.ncbi.nlm.nih.gov/snp/rs879254854) | St. Petersburg | Zakharova et al., 2005, 2007 | None |
| 9 | c.1303delG | g.29014del | 11113393 | p.(Glu435fs)  [p.(Glu435Argfs*16), FsE414:M429X] | CD132146  Disease causing mutation | 1 | Pathogenic  Accession:  VCV 000251776.1 | [rs879254857](https://www.ncbi.nlm.nih.gov/snp/rs879254857) | St. Petersburg | Zakharova et al., 2005, 2007 | Germany |
| 11 | c.1618delG | g.31745del | 11116125 | p.(Ala540fs)  [p.(Ala540Profs*8), A519PfsX8] | CD099865  Disease causing mutation | 1 | N/A | N/A | Moscow | Meshkov et al., 2009 | None |
| 11 | c.1686_1693delinsT | g.31813_31820delinsT | 11116193-11116200 | p.(Trp562fs)  [p.(Trp562Cysfs*5), FsW541:L547X] | CX1313040  Disease causing mutation | 1 | Pathogenic  Accession: VCV 000251968.1 | [rs879254984](https://www.ncbi.nlm.nih.gov/snp/rs879254984) | Petrozavodsk | Komarova et al., 2013c | None |
| 13 | c.1855_1856insA | g.35721_ 35722insA | 11120101-11120102 | p.(Phe619fs)  [p.(Phe619Tyrfs*26)  FsV597:A622X] | CI051352  Disease causing mutation | 1 | Pathogenic  Accession: VCV 000252082.1 | [rs879255053](https://www.ncbi.nlm.nih.gov/snp/rs879255053) | St. Petersburg | Zakharova et al., 2005, 2007 | None |
| 15 | c.2191delG | g.38844del | 11123223 | p.(Val731fs)  [p.(Val731Serfs*6), FsV710:V715X] | CD136949  Disease causing mutation | 1 | Pathogenic  Accession: VCV 000252253.1 | [rs879255161](https://www.ncbi.nlm.nih.gov/snp/rs879255161) | Petrozavodsk | Komarova et al., 2013 b,c  Korneva et al., 2014 | None |
| 17 | c.2416dupG | [g.45159dup](https://www.ncbi.nlm.nih.gov/nuccore/NG_009060.1?report=graph&mk=45159\|NG_009060.1\:g.45159dup\|green) | 11129534-11129535 | p.(Val806fs)  [=p.Val806Gly  fs*11)] | N/A  Likely pathogenic | 1 | Conflicting interpretations of pathogenicity​  Likely pathogenic Pathogenic;  Uncertain significance  VCV  000252330.11 | N/A | Moscow | Semenova et al., 2020 | USA, Europe |
| **Missense mutations** | | | | | | | | | | | |
| 4 | c.326G>A  TGC>TAC | g.20852G>A | 11105232 | p.(Cys109Tyr)  [C88Y] | CM983629  Disease causing mutation | 1 | Pathogenic/ Likely pathogenic  Accession: VCV  000226319.3 | [rs121908042](https://www.ncbi.nlm.nih.gov/snp/rs121908042) | Moscow | Meshkov et al., 2009 | Great Britain, Poland |
| 4 | c.444T>G  TGT>TGG | g.20970T>G | 11105350 | p.(Cys148Trp)  [C127W] | CM981179  Disease causing mutation | 1 | Likely pathogenic  Accession:  VCV  000251228.1 | [rs879254528](https://www.ncbi.nlm.nih.gov/snp/rs879254528) | St. Petersburg | Chakir et al., 1998a | None |
| 4 | c.451G>C  GCC>CCC | g.20977G>C | 11105357 | p.(Ala151Pro)  [A130P] | CM014574  Disease causing mutation | 1 | Variant of uncertain significance  Accession: VCV  000251234.3 | [rs763233960](https://www.ncbi.nlm.nih.gov/snp/rs763233960) | St. Petersburg | Tatishcheva et al., 2001; Zakharova et al., 2007 | The Netherlands |
| 4 | c.478T>G TGC>GGC | g.21004T>G | 11105384 | p.(Cys160Gly)  [=C160G, C139G] | CM981180  Disease causing mutation | 4 | Likely pathogenic Accession: VCV  000251248.1 | [rs879254540](https://www.ncbi.nlm.nih.gov/snp/rs879254540) | St. Petersburg, Novosibirsk, Moscow | Chakir et al., 1998а; Mandelshtam and Maslennikov, 2001; Meshkov et al., 2004, 2009 | None |
| 4 | c.499T>C  TGC>CGC | g.21025T>C | 11105405 | p.(Cys167Arg)  [C146R] | CM014575  Disease causing mutation | 1 | Likely pathogenic  Accession: VCV  000251255.1 | [rs879254547](https://www.ncbi.nlm.nih.gov/snp/rs879254547) | St. Petersburg | Tatishcheva et al., 2001; Zakharova et al., 2007 | None |
| 4 | c.530C>T  TCG>TTG | g.21056C>T | 11105436 | p.(Ser177Leu)  [FH Puerto Rico] | CM890079  Disease causing mutation | 1 | Pathogenic./Likely pathogenic  Accession:  VCV000003686.8 | N/A | Moscow | Semenova et al., 2020 | Europe, Latin America, USA |
| 4 | c.534T>G  GAT>GAG | g.21060T>G | 11105440 | p.(Asp178Glu)  [D178E] | CM003319  Disease causing mutation | N/A | Pathogenic/  Likely pathogenic  Accession: VCV  000251287.1 | [rs879254566](https://www.ncbi.nlm.nih.gov/snp/rs879254566) | Novosibirsk | Shakhtshneider et al., 2019a | Austria, Germany, France |
| 4 | c.542C>T  CCG>CTG | g.21068C>T | 11105448 | p.(Pro181Leu) | CM150269  Disease causing mutation | 1 | Likely pathogenic  Accession:  RCV000497065.3 | N/A | Moscow | Semenova et al., 2020 | Brazil |
| 4 | c.551G>A  TGT>TAT | g.21077G>A | 11105457 | p.(Cys184Tyr) [C163Y] | CM981182  Disease causing mutation | 1 | Pathogenic/ Likely pathogenic  Accession: VCV  000003739.5 | [rs121908039](https://www.ncbi.nlm.nih.gov/snp/rs121908039) | Moscow | Meshkov et al., 2009 | Brazil, Canada, Great Britain, Ireland, Portugal, Scotland |
| 4 | c.601G>A  GAG>AAG | g.21127G>A | 11105507 | p.(Glu201Lys) [E180K] | CM067689  Disease causing mutation | 1 | Likely pathogenic  Accession: VCV  000251317.1 | [rs879254589](https://www.ncbi.nlm.nih.gov/snp/rs879254589) | Moscow | Meshkov et al., 2009 | Malaysia, Venezuela |
| 4 | c.622G>A  GAG>AAG | g.21148G>A | 11105528 | p.(Glu208Lys)  [E187K] | CM920419  Disease causing mutation | 2 | Pathogenic/ Likely pathogenic  Accession: VCV  000251328.2 | [rs879254597](https://www.ncbi.nlm.nih.gov/snp/rs879254597) | St. Petersburg, Moscow | Tatishcheva et al., 2001; Zakharova et al., 2007; Averkova et al., 2018 | Czech Republic, Israel, The Netherlands, South Africa, Sweden, etc. |
| 4 | c.626G>A  TGC>TAC | g.21152G>A | 11105532 | p.(Cys209Tyr)  [C209Y, C188Y] | CM012621  Disease causing mutation | 1 | Pathogenic/ Likely pathogenic  Accession:  VCV  000251332.1 | [rs879254600](https://www.ncbi.nlm.nih.gov/snp/rs879254600) | St. Petersburg | Tatishcheva et al., 2001; Zakharova et al., 2007 | Czech Republic |
| 4 | c.661G>T  GAC>TAC | g.21187G>T | 11105567 | p.(Asp221Tyr)[D221Y, D200Y] | CM950754  Disease causing mutation | 1 | Pathogenic/ Likely pathogenic  Accession: VCV  000251356.1 | [rs875989906](https://www.ncbi.nlm.nih.gov/snp/rs875989906) | Moscow | Meshkov et al., 2009 | Germany, Finland, RSA Indians, Italy, Portugal, Spain, etc. |
| 4 | c.682G>A  GAG>AAG | g.21208G>A | 11105588 | p.(Glu228Lys) [E228K, E207K] | CM900153  Disease causing mutation | 1 | Pathogenic/ Likely pathogenic Accession: VCV  000003691.7 | [rs121908029](https://www.ncbi.nlm.nih.gov/snp/rs121908029) | Moscow | Meshkov et al., 2009 | French Canadian, China, Israel, Korea, Mexico, The Netherlands, Portugal, etc. |
| 5 | c.782G>T  TGC>TTC | g.22272G>T | 11106652 | p.(Cys261Phe)  [C261F] | CM990795  Disease causing mutation | N/A | Likely pathogenic  Accession: VCV  000003740.4 | [rs121908040](https://www.ncbi.nlm.nih.gov/snp/rs121908040) | Novosibirsk | Voevoda et al., 2008 | Denmark, Norway, Sweden |
| 5 | c.796G>A  GAT>AAT | g.22286G>A | 11106666 | p.(Asp266Asn)  [D266N]] | CM013562  Disease causing mutation | N/A | Pathogenic/ Likely pathogenic  Accession: VCV  000226334.1 | [rs875989907](https://www.ncbi.nlm.nih.gov/snp/rs875989907) | Novosibirsk | Shakhtshneider et al., 2019a | Czech Republic, Denmark, Japan, The Netherlands, Norway, Tunis, etc. |
| 6 | c.862G>A  GAA>AAA | g.23056G>A | 11107436 | p.(Glu288Lys)  [E288K] | CM990796  Disease causing mutation | N/A | Conflicting interpretations of pathogenicity  Accession: VCV  000161268.4 | [rs368657165](https://www.ncbi.nlm.nih.gov/snp/rs368657165) | Novosibirsk | Voevoda et al., 2008 | Germany Italy, The Netherlands, New Zealand, Portugal |
| 6 | [c.922G>A](https://www.ncbi.nlm.nih.gov/nuccore/NM_000527.4?report=graph&mk=922\|NM_000527.4\:c.922G%3EA\|green)  GAA>AAA | [g.23116G>A](https://www.ncbi.nlm.nih.gov/nuccore/NG_009060.1?report=graph&mk=23116\|NG_009060.1\:g.23116G%3EA\|green) | 11107496 | p.(Glu308Lys) | CM066911  Disease causing mutation | 1 | Likely pathogenic  Accession:  VCV000251528.1 | [rs879254721](https://www.ncbi.nlm.nih.gov/snp/rs879254721) | Moscow | Semenova et al., 2020 | Poland, the Netherlands |
| 7 | c.973T>C  TGT>CGT | g.26304T>C | 11110684 | p.(Cys325Arg)  [C325R] | N/A | N/A | Likely pathogenic  Accession: VCV  000627979.1 | [rs1568600328](https://www.ncbi.nlm.nih.gov/snp/rs1568600328) | Novosibirsk | Shakhtshneider et al., 2019a | None |
| 7 | c.986G>A  TGC>TAC | g.26317G>A | 11110697 | p.(Cys329Tyr)  [=C329Y, C308Y] | CM981186  Disease causing mutation | 5 | Conflicting interpretations of pathogenicity  Accession:  VCV  000226344.2 | [rs761954844](https://www.ncbi.nlm.nih.gov/snp/rs761954844) | St. Petersburg, Novosibirsk | Zakharova et al., 2005, 2007; Shakhtshneider et al., 2019a; Semenova et al., 2020 | Canada, Chinese, Czech Republic, Filipins, Poland, Taiwan, The Netherlands |
| 7 | c.1009G>A  GAG>AAG | g.26340G>A | 11110720 | p.(Glu337Lys)  [E337K] | CM182772  Disease causing mutation? | 1 | Variant of uncertain significance Accession: VCV  000523729.1 | [rs539080792](https://www.ncbi.nlm.nih.gov/snp/rs539080792) | Novosibirsk | Shakhtshneider et al., 2019a | Chile, Singapore |
| 7 | c.1019G>A  TGC>TAC | g.26350G>A | 11110730 | p.(Cys340Tyr)  [C340Y] | CM042078  Disease causing mutation | 1 | Conflicting interpretations of pathogenicity  Accession: VCV  000251600.2 | [rs755757866](https://www.ncbi.nlm.nih.gov/snp/rs755757866) | Novosibirsk | Shakhtshneider et al., 2019a | Spain |
| 7 | c.1054T>C  TGC>CGC | [g.26385T>C](https://www.ncbi.nlm.nih.gov/nuccore/NG_009060.1?report=graph&mk=26385\|NG_009060.1\:g.26385T%3EC\|green) | 11110765 | p.(Cys352Arg) | CM984574 | 2 | Likely pathogenic  Accession:  VCV000251618.1 | [rs879254769](https://www.ncbi.nlm.nih.gov/snp/rs879254769) | Moscow | Semenova et al., 2020 | Austria |
| 9 | c.1202T>A  CTC>CAC | g.28913T>A | 11113293 | p.(Leu401His)  [L401H, L380H, FH Pori; FH Finn-7] | CM950762  Disease causing mutation | 2 | Likely pathogenic  Accession:  VCV  000003735.1 | [rs121908038](https://www.ncbi.nlm.nih.gov/snp/rs121908038) | St. Petersburg, Novosibirsk | Zakharova et al., 2005; Shakhtshneider et al., 2019a | Finland, The Netherlands |
| 9 | c.1228A>G  AGG>GGG | g.28939A>G | 11113319 | p.(Arg410Gly)[R410G] | N/A  Likely pathogenic | 1 | N/A | N/A | Novosibirsk | Voevoda et al., 2008 | None |
| 9 | c.1234A>G  ATG>GTG | g.28945A>G | 11113325 | p.(Met412Val) [M412V] | N/A  Likely pathogenic | 1 | N/A | [rs1225797407](https://www.ncbi.nlm.nih.gov/snp/rs1225797407)  Allele G of this SNP not described | Novosibirsk | Voevoda et al., 2008 | None |
| 9 | c.1246C>T CGG>TGG | g.28957C>T | 11113337 | p.(Arg416Trp)[R416W, R395W] | CM970893  Disease causing mutation | 4 | Pathogenic/ Likely pathogenic  Accession: VCV  000183110.4 | [rs570942190](https://www.ncbi.nlm.nih.gov/snp/rs570942190) | Moscow, Novosibirsk | Meshkov et al., 2009; Shakhtshneider et al., 2019a; Semenova et al., 2020 | Austria, Canada, Czech Republic Germany, Israel, Italy, Korea, The Netherlands, Norway, Poland, Spain, Taiwan, etc. |
| 9 | c.1253A>G  GAG>GGG | g.28964A>G | 11113344 | p.(Glu418Gly) [E418G] | CM172606  Disease causing mutation | N/A | N/A | N/A | Novosibirsk | Voevoda et al., 2008 | China |
| 9 | c.1295T>G  CTG>CGG | g.29006T>G | 11113386 | p.(Leu432Arg) [L432R] | N/A  Likely pathogenic | 1 | N/A | [rs879254855](https://www.ncbi.nlm.nih.gov/snp/rs879254855) Allele G of this SNP not described | Novosibirsk | Voevoda et al., 2008 | Other populations, no citations available |
| 9 | c.1299C>G  GAC>GAG | g.29010C>G | 11113390 | p.(Asp433Glu) [D433E] | N/A  Likely pathogenic | 1 | N/A | [rs778309692](https://www.ncbi.nlm.nih.gov/snp/rs778309692) | Novosibirsk | Voevoda et al., 2008 | Rare in other populations, no citations available. |
| 9 | c.1322T>A  ATC>AAC | g.29033T>A | 11113413 | p.(Ile441Asn)  [I441N, I420N] | CM920454  Disease causing mutation | 1 | Likely pathogenic  Accession: VCV  000251782.1 | [rs879254862](https://www.ncbi.nlm.nih.gov/snp/rs879254862) | Russia | Hobbs et al., 1992 | Greece |
| 9 | c.1327T>C  TGG>CGG | g.29038T>C | 11113418 | p.(Trp443Arg) [W443R, W422R] | CM045800 Disease causing mutation | 2 | N/A | [rs773566855](https://www.ncbi.nlm.nih.gov/snp/rs773566855) | Moscow, Petrozavodsk | Meshkov et al., 2004, 2009; Korneva et al., 2017a,b; Semenova et al., 2020 | Rare in other populations, no citations available |
| 10 | c.1444G>T GAC>TAC | g.29240G>T | 11113620 | p.(Asp482Tyr)  [D482Y, D461Y] | CM045709  Disease causing mutation | 1 | Likely pathogenic Accession: VCV  000251845.1 | [rs139624145](https://www.ncbi.nlm.nih.gov/snp/rs139624145) | Moscow | Krapivner et al., 2001; Meshkov et al., 2004, 2009 | None |
| 10 | c.1465T>A  TAC>AAC | g.29261T>A | 11113641 | p.(Tyr489Asn) | N/A  Likely pathogenic? | 1 | N/A | N/A | Moscow | Semenova et al., 2020 | None |
| 11 | c.1633G>A  GGG>AGG | g.31760G>C | 11116140 | p.(Gly545Arg) | CM108002 | 1 | Likely pathogenic  Accession:  RCV000237535.3 | [rs879254965](https://www.ncbi.nlm.nih.gov/variation/tools/1000genomes/?chr=19&from=11226816&to=11226816&gts=rs879254965&mk=11226816:11226816\|rs879254965) | Moscow | Semenova et al., 2020 | Brazil, France, Korea |
| 11 | c.1672G>A  GAA>AAA | g.31799G>A | 11116179 | p.(Glu558Lys)  [E558K] | N/A  Likely pathogenic? | 1 | N/A | N/A | Novosibirsk | Voevoda et al., 2008 | None |
| 12 | c.1741A>C  AAA>CAA | g.32514A>C | 11116894 | p.(Lys581Gln) | N/A | 1 | N/A | N/A | Moscow | Semenova et al., 2020 | None |
| 12 | c.1756T>C  TCA>CCA | g.32529T>C | 11116909 | p.(Ser586Pro) | Likely pathogenic? | 2 | N/A | N/A | Moscow | Semenova et al., 2020 | None |
| 12 | c.1775G>A  GGG>GAG | g.32548G>A | 11116928 | p.(Gly592Glu)  [G592E, G571E, FH Sicily;  FH Foggia-1;  FH Naples4;  FH Sicilia-4] | CM920464  Disease causing mutation | 8 | Pathogenic/ Likely pathogenic  Accession: VCV  000161271.6 | [rs137929307](https://www.ncbi.nlm.nih.gov/snp/rs137929307) | St. Petersburg, Novosibirsk | Zakharova et al., 2001, 2007; Voevoda et al., 2008, etc.; Semenova et al., 2020 | Austria, Belgium, Brazil, Canada, Greece, Czech Republic, Germany, Italy, The Netherlands, Norway, Poland, Portugal, Spain, USA, etc. |
| 12 | c.1813C>G  CTG>GTG | g.32586C>G | 11116966 | p.(Leu605Val)  [L605V] | N/A | 1 | N/A | [rs879255031](https://www.ncbi.nlm.nih.gov/snp/rs879255031)  Allele G of this SNP not described | Novosibirsk | Voevoda et al., 2008 | None |
| 12 | c.1814T>G  CTG>CGG | g.32587T>G | 11116967 | p.(Leu605Arg)  [L605R] | N/A  Likely pathogenic | 1 | N/A | [rs875989932](https://www.ncbi.nlm.nih.gov/snp/rs875989932)  Allele G of this SNP not described | Novosibirsk | Voevoda et al., 2008 | None |
| 12 | c.1835C>G  GCC>GGC | g.32608C>G | 11116988 | p.(Ala612Gly)  A612G | N/A  Likely pathogenic | 1 | N/A | [rs377449975](https://www.ncbi.nlm.nih.gov/snp/rs377449975)  Allele G of this SNP not described | Novosibirsk | Voevoda et al., 2008 | None |
| 13 | c.1864G>A  GAT>AAT | g.35730G>A | 11120110 | p.(Asp622Asn)  [D622N, D601N] | CM051100  Disease causing mutation | 1 | Pathogenic/ Likely pathogenic  Accession:  VCV  000252092.2 | [rs879255059](https://www.ncbi.nlm.nih.gov/snp/rs879255059) | St. Petersburg | Zakharova et al., 2005, 2007 | Czech Republic, China |
| 14 | c.1999T>A  TGT>AGT | g.36001T>A | 11120381 | p.(Cys667Ser)  [C667S, C646S] | CM051101  Disease causing mutation | 1 | Likely pathogenic  Accession: VCV  000252162.1 | [rs150021927](https://www.ncbi.nlm.nih.gov/snp/rs150021927) | St. Petersburg | Zakharova et al., 2005, 2007 | None |
| 15 | c.2155G>C  GTG>CTG | g.38808G>C | 11123188 | p.(Val719Leu)  [V719L, V698L] | CM045799  Disease causing mutation | 1 | Likely benign  Accession: VCV  000252243.1 | [rs879255151](https://www.ncbi.nlm.nih.gov/snp/rs879255151) | Moscow | Meshkov et al., 2004, 2009 | Unknown |
| 16 | c.2326G>T  GCT>TCT | g.43642G>T | 11128022 | p.(Ala776Ser)  [A776S] | N/A | 1 | N/A  Variant of uncertain significance | N/A | Moscow | Averkova et al., 2018 | None |
| 16 | c.2374A>T  ATT>TTT | g.43690A>T | 11128070 | p.(Ile792Phe)  [I792F, I771F, FH Russia-2] | CM920470  Disease causing mutation | 1 | Conflicting interpretations of pathogenicity  Accession: VCV  000252294.1 | [rs761123215](https://www.ncbi.nlm.nih.gov/snp/rs761123215) | Russia | Hobbs et al., 1992 | Unknown |
| 16 | c.2389G>A  GTG>ATG | g.43705G>A | 11128085 | p.(Val797Met)  [V797M, V776M] | CM950772  Disease causing mutation | 1 | Conflicting interpretations of pathogenicity  Accession:  VCV  000226393.5 | [rs750518671](https://www.ncbi.nlm.nih.gov/snp/rs750518671) | St. Petersburg | Zakharova et al., 2005, 2007 | China, Cuba, Czech Republic, France, India, Italy, Japan, Norway, Mexico, The Netherlands, Portugal, Scotland South Africa |
| 17 | c.2479G>A GTC>ATC | g.45222G>A | 11129602 | p.(Val827Ile)[V827I, V806I, FH New York-5] | CM920471Disease causing mutation? | N/A | Conflicting interpretations of pathogenicity Accession: VCV000036462.8 | [rs137853964](https://www.ncbi.nlm.nih.gov/snp/rs137853964) | St. Petersburg, Novosibirsk | Zakharova et al., 2005, 2007; Shakhtshneider et al., 2019a | Czech Republic, Denmark, Germany, Israel, Italy, The Netherlands, Norway, Slovak Republic, USA, etc. |
| **In-frame deletions and duplications** | | | | | | | | | | | |
| 3 | c.191_313del | g.18254_18376del | 11102634_11102656 | p.(Leu64_Pro105delinsSer) | N/A | 1 | N/A | N/A | Moscow | Semenova et al., 2020 | France, The Netherlands |
| 4 | c.347_349del | g.20873_20875del | 11105253-11105255 | p.(Cys116_His117delinsTyr) [347delGCC] | CD984000  Disease causing mutation | 1 | Likely pathogenic  Accession: VCV  000251164.1 | [rs879254483](https://www.ncbi.nlm.nih.gov/snp/rs879254483) | St. Petersburg | Chakir et al., 1998b | None |
| 4 | c.654-656 delTGG  Cited also as 652-654delGGT or 651-653del3 | g.21177_21179delTGG | 11105560-11105562 | p.(Gly219del)  [G219del,  G218del, G197del, deltaG197, dG197, FH Lithuania, FH Piscataway] | CD910540  Disease causing mutation | 2 | Pathogenic/ Likely pathogenic  Accession: VCV  000226329.6 | [rs121908027](https://www.ncbi.nlm.nih.gov/snp/rs121908027) | St. Petersburg, Novosibirsk | Mandelshtam et al., 1998; Mandelshtam and Maslennikov, 2001; Zakharova et al., 2005, 2007 | Czech Republic, Germany, Great Britain, Israel, The Netherlands, Poland, South Africa, USA, usually in Ashkenazi Jews |
| 4 | c.658_663del | g.21184_21189del | 11105564-11105569 | p.(Pro220_Asp221del)  [P199_D200del] | CD099863  Disease causing mutation | 2 | Pathogenic Accession: VCV  000440589.1 | [rs1555803409](https://www.ncbi.nlm.nih.gov/snp/rs1555803409) | Moscow | Meshkov et al., 2009; Semenova et al., 2020 | None |
| 4 | c.673_681dup | g.21199_21207dup | 11105579-11105587 | p.(Lys225_Asp227dup)  [D203S205dup] | CI099862  Disease causing mutation | 1 | Likely pathogenic Accession: VCV  000251377.1 | [rs1555803425](https://www.ncbi.nlm.nih.gov/snp/rs1555803425) | Moscow | Meshkov et al., 2004, 2009 | France |
| 4 | c.672_686del Originally described as c.670_684del | g.21198_21212del | 11105578-11105592 | p.(Asp224_ Glu228del)  [D203_E207del] | CD099864  Disease causing mutation | 1 | Likely pathogenic Accession: VCV  000441189.1 | [rs1555803439](https://www.ncbi.nlm.nih.gov/snp/rs1555803439) | Moscow | Meshkov et al., 2009 | None |
| 11 | N/A | N/A | Originally described as chr19: 11226838-11226855GRCh37 | Originally described as 17 bp deletion. | N/A  Disease causing mutation? | 1 | N/A | N/A | Novosibirsk | Shakhtshneider et al., 2019a  Description in abstract ambiguous. | None |
| **Splice site mutations** | | | | | | | | | | | |
| 3i | c.313+1G>A | g.18407G>A | 11102787 | Exon 3 skip  [IVS3+1 G>A, FH-Elverum,  FH Olbia] | CS941506  Disease causing mutation | 2 | Conflicting interpretations of pathogenicity  Accession: VCV  000003736.6 | [rs112029328](https://www.ncbi.nlm.nih.gov/snp/rs112029328) | St. Petersburg | Zakharova et al., 2005, 2007 | Austria, Belgium, Denmark, Germany, Great Britain, Italy, Korea, The Netherlands, Norway, South Africa (black), Spain, Sweden |
| 3i | c.313+2T>G | g.18408T>G | 11102788 | Intron splicing defect [IVS3+2T>G] | N/A | 1 | N/A | [rs793888517](https://www.ncbi.nlm.nih.gov/snp/rs793888517)  Allele G not described | Petrozavodsk | Korneva et al., 2016; Korneva et al., 2017b | None |
| 6i | c.940+3_940+6delGAGT | g.23137_23140del | 11107517-11107520 | Intron splicing defect [IVS6+3_6del] | N/A | 2 | N/A | N/A | Moscow | Semenova et al., 2020 | None |
| 9i | c.1358+1G>A | g.29070G>A | 11113450 | Intron splicing defect [IVS9+1G>A] | CS930843  Disease causing mutation | 1 | Pathogenic/ Likely pathogenic  Accession: VCV 000251802.1 | [rs775924858](https://www.ncbi.nlm.nih.gov/snp/rs775924858) | St. Petersburg | Zakharova et al., 2005, 2007 | The Netherlands |
| 12i | c.1846-3T>G | g.35709T>G | 11117089 | Intron splicing defect [IVS12-3T>G] | N/A  Likely pathogenic | 1 | N/A | N/A | Moscow | Semenova et al., 2020 | None |
| 16i | c.2389+5G>A | g.43710G>A | 11128090 | Intron splicing defect  [IVS9+5G>A] | CS067833  Disease causing mutation | 3 | Conflicting interpretations of pathogenicity Accession: VCV 000252306.2 | [rs879255191](https://www.ncbi.nlm.nih.gov/snp/rs879255191) | Moscow, Novosibirsk | Meshkov et al., 2009; Shakhtshneider et al., 2017; Semenova et al., 2020 | Italy, Poland |
| **Gross insertions and deletions** | | | | | | | | | | | |
| 3i_6i | Not yet available | N/A | 5 kb deletion characterized at genomic level | Not yet available | CG931314  Disease causing mutation | 1 | N/A | N/A | St. Petersburg | [Mandelshtam et al., 1993](http://www.ncbi.nlm.nih.gov/pubmed/08401534) | None |
| 4-8 | c.314-?_1186 + ?dup | g.20840-?_27259+? | N/A | p.(Pro106_Val395dup)  Inframe duplication of exons 4 to 8 | N/A | 1 | N/A | N/A | Moscow | Semenova et al., 2020 | Poland, Czech Republic, The Netherlands |
| 5i_6i | c.817+303_  940+943del | g.22610_24077del | 11106990-11108457 | p.(Val273_  Cys313del)  In frame 1468 bp deletion of exon 6 | N/A  Disease causing mutation? | 1 | N/A | N/A | Moscow, identified by target sequencing | Averkova et al., 2018 | None |
| 14i-16i | c.2141-966_2390-330del | g.37256_44815del | 111121636-111129195 | p.(Glu714_Ile796del)  Deletion of exons 15 and 16 | N/A  Disease causing mutation? | 1 | N/A | N/A | Moscow | Semenova et al., 2020 | Japan, Brazil |
