# Supplementary material for: Familial Hypercholesterolemia in Russia: Three Decades of Genetic Studies
Source: Front Genet. 2020 Dec 17;11:550591. doi: 10.3389/fgene.2020.550591 (PMC7773754; doi:10.3389/fgene.2020.550591)
Supplement: Supplementary file 2 [file Table_2.docx]

**Supplementary Table 2. Neutral polymorphisms and silent mutations detected in the *LDLR* gene in Russia**

| Exon No. | NM_  000527.4 (Nucleotide substitution  LDLR) | HGVS nomenclature NG_ 009060.1 (LDLR)  (LRG 274) | Genomic location GRCh38 (Chr19) p12 | Predicted effect on protein | HGMD Public Accession | Common(>1% of alleles) or rare | ClinVar estimate of pathogenicity, Accession | dbSNP rs number | Populations in Russia | References for Russia | Other populations in the world |
| --- | --- | --- | --- | --- | --- | --- | --- | --- | --- | --- | --- |
| 2 | c.58G>A  GGG>GAG | g.5226G>A | 11089606 | p.(Gly20Arg)  [G20R,  G(-2)R] | CM022807 Disease causing mutation? | Rare | Conflicting interpretations of pathogenicity, likely benign  Accession: VCV  000161272.4 | [rs147509697](https://www.ncbi.nlm.nih.gov/snp/rs147509697) | Petrozavodsk, Moscow | Komarova et al., 2015с; Averkova et al., 2018 | Austria, Croatia, France, Latvia, The Netherlands, New Zealand, Slovakia, Turkey |
| 2 | c.81C>T  TGC>TGT | g.15856C>T | 11100236 | p.(Cys27=)  [C6C, C27C *SfaNI* RFLP] | N/A | Common | Benign/Likely benign  Accession: VCV  000251010.2 | [rs2228671](https://www.ncbi.nlm.nih.gov/snp/rs2228671) | St. Petersburg, Novosibirsk, Moscow, Petrozavodsk | Zakharova et al., 2005, 2007; Meshkov et al., 2009; Voevoda et al., 2008; Komarova, 2013b | China, Great Britain, Greece, Italy, Morocco, Southern Europe |
| 4 | c.447T>C  GGT>GGC | g.20973T>C | 11105353 | p.(.Gly149=)  [G149G, G128G] | N/A | Rare | Benign/Likely benign  Accession: VCV  000251230.3 | [rs765386678](https://www.ncbi.nlm.nih.gov/snp/rs765386678) | St. Petersburg | Tatishcheva et al., 2001;  Zakharova et al., 2007 | Other populations, very rare, citations unavailable |
| 5 | c.750T>C  CAT>CAC | g.22240T>C | 11106620 | p.(His250=)  [H250H, H229H] | N/A | Rare | Likely benign  Accession: VCV  000251433.1 | [rs141929940](https://www.ncbi.nlm.nih.gov/snp/rs141929940) | St. Petersburg | Zakharova et al., 2001, 2007 | Other populations, very rare, citations unavailable |
| 8 | c.1171G>A  GCC>ACC | g.27244G>A | 11111624 | p.(Ala391Thr)  [A391T, A370T, *StuI* RFLP] | CM014366  Disease-associated polymorphism | Common | Benign/Likely benign Accession: VCV  000183138.3 | [rs11669576](https://www.ncbi.nlm.nih.gov/snp/rs11669576) | St. Petersburg, Moscow, Novosibirsk, Petrozavodsk | Zakharova et al., 2001, 2007; Meshkov et al., 2009, Voevoda et al., 2008; Komarova et al., 2013b | Denmark, Germany, Great Britain, France, Poland, South Africa, etc. |
| 9 | c.1194C>T  ATC>ATT | g.28905C>T | 11113285 | p.(Ile398=)  [I398I,  I377I] | CM071836  Disease causing mutation? | Common | Conflicting interpretations of pathogenicity  Accession: VCV  000224620.2 | [rs13306498](https://www.ncbi.nlm.nih.gov/snp/rs13306498) | Petrozavodsk | Komarova et al., 2013c | Austria, France |
| 10 | c.1413A>G  AGA>AGG | g.29209A>G | 11113589 | p.(Arg471=)  [R471R,  R450R] | N/A | Common | Benign/Likely benign  Accession: VCV  000200916.2 | [rs5930](https://www.ncbi.nlm.nih.gov/snp/rs5930) | St. Petersburg, Petrozavodsk | Tatishcheva et al., 2001; Zakharova et al., 2007; Komarova et al., 2013c | Asia, Brazil, Turkey |
| 10 | c.1545C>T  AAC>AAT | g.29341C>T | 11113721 | p.(Asn515=)  [N515N, N494N] | N/A | Common | Benign/Likely benign  Accession:  VCV 000251891.3 | [rs147896205](https://www.ncbi.nlm.nih.gov/snp/rs147896205) | St. Petersburg | Tatishcheva et al., 2001; Zakharova et al., 2007 | Estonia, Poland, Sweden |
| 11 | c.1617C>T  CCC>CCT | g.31744C>T | 11116124 | p.(Pro539=)  [P539P, P518P] | N/A | Common | Benign/Likely benign Accession: VCV 000251936.2 | [rs5929](https://www.ncbi.nlm.nih.gov/snp/rs5929) | St. Petersburg, Moscow, Petrozavodsk | Zakharova et al., 2005, 2007; Meshkov et al., 2009; Komarova et al., 2013c | China, Malaysia, Morocco, Taiwan |
| 11 | c.1647T>N  GGT>GGN  Original description ambiguous | g.31774T>N | 11116154 | p.(Gly549=)  [G549G] | N/A | N/A | N/A  Probably benign | N/A | Novosibirsk | Voevoda et al., 2008 | None |
| 11 | c.1702C>T  CTA>TTA | g.31829C>T | 11116209 | p.(Leu568=)  [L568L] | N/A | N/A | N/A  Probably benign | [rs746959386](https://www.ncbi.nlm.nih.gov/snp/rs746959386) | Novosibirsk | Voevoda et al., 2008 | Other populations, very rare, citations unavailable |
| 12 | c.1728C>T  TAC>TAT | g.32501C>T | 11116881 | p.(Tyr576=)  [Y576Y] | N/A | N/A | N/A  Probably benign | N/A | Novosibirsk | Voevoda et al., 2008 | None |
| 12 | c.1773C>T  AAC>AAT  c. 1773 T/C | g.32546C>T | 11116926 | p.(Asn591=)  [N591N, N570N] | CM984053  Associated with increased plasma LDL cholesterol. | Common | Benign/Likely benign  Accession: VCV 000200917.2 | [rs688](https://www.ncbi.nlm.nih.gov/snp/rs688) | St. Petersburg, Novosibirsk, Petrozavodsk | Zakharova et al., 2001, 2007; Voevoda et al., 2008; Komarova et al., 2013c | Brazil, China, Morocco, Northern Italy, The Netherlands, US |
| 13 | c.1920C>T  AAC>AAT | g.35786C>T | 11120166 | p.(Asn640=)  [N640N, N619N] | CM071834  Disease causing mutation? | Rare? | Conflicting interpretations of pathogenicity  Accession: VCV 000237869.3 | [rs5926](https://www.ncbi.nlm.nih.gov/snp/rs5926) | Petrozavodsk | Komarova et al., 2013bc | Austria, Poland, Portugal, Spain |
| 13 | c.1959T>C  GTT>GTC | g.35825T>C | 11120205 | p.(Val653=)  [V653V, V632V, *AvaII* RFLP] | CM080431  Increased plasma LDL cholesterol. | Common | Benign/Likely benign  Accession: VCV 000252131.3 | [rs5925](https://www.ncbi.nlm.nih.gov/snp/rs5925) | St. Petersburg, Novosibirsk, Petrozavodsk | Zakharova et al., 2007; Voevoda et al., 2008 Komarova et al., 2013c | Finland, Malaysia, Morocco, Poland, The Netherlands, etc. |
| 15 | с.2232A>G  CGG>CGA | g.38885A>G | 11123265 | p.(Arg744=)  [R744R,  R723R, originally erroneously cited as R730R] | N/A | Common | Benign/Likely benign  Accession: VCV 000252262.2 | [rs5927](https://www.ncbi.nlm.nih.gov/snp/rs5927) | St. Petersburg, Petrozavodsk | Zakharova et al., 2007; Komarova et al., 2013b,c | Asia, China, Germany, Malaysia |
